# Supplementary material for: The interactome of intact mitochondria by cross-linking mass spectrometry provides evidence for coexisting respiratory supercomplexes
Source: Mol Cell Proteomics. 2017 Dec 8;17(2):216–32. doi: 10.1074/mcp.RA117.000470 (PMC5795388; doi:10.1074/mcp.RA117.000470)
Supplement: Supplemental Data [file supp_RA117.000470_133922_0_supp_23978_fzffwf.zip › spectra_annotation/mito_spectra_annotation/164-1-18-1-13-1.pdf]

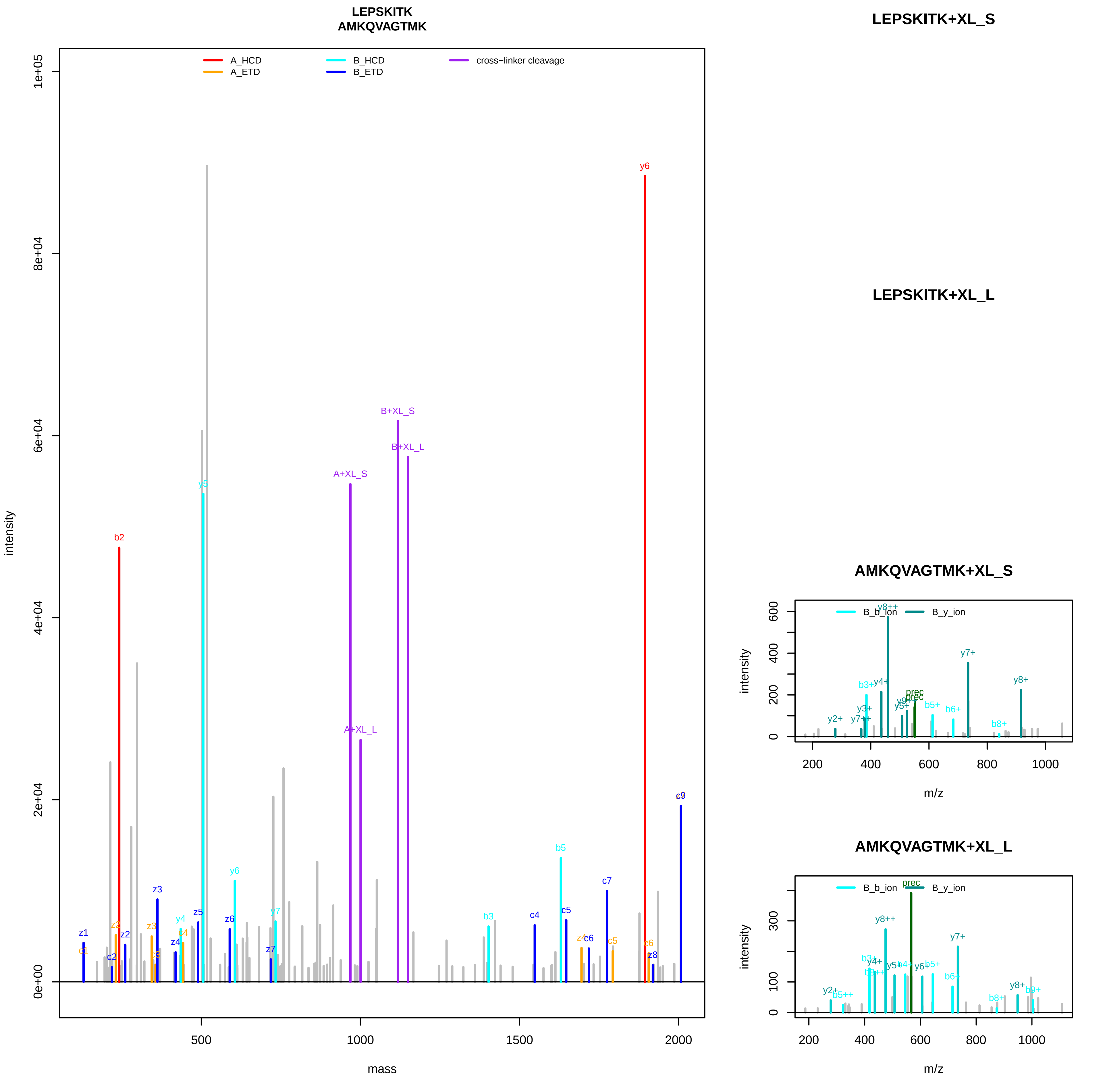

LEPSKITK
AMKQVAGTMK
A_HCD
A_ETD
B_HCD
B_ETD
cross−linker cleavage
intensity
mass
0e+00
2e+04
4e+04
6e+04
8e+04
1e+05
500
1000
1500
2000
y6
b2
B+XL_S
B+XL_L
A+XL_S
A+XL_L
y5
c9
b5
y6
c7
z1
z2
z3
z4
z5
z6
z7
z8
c2
c4
c5
c6
y4
y7
b3
LEPSKITK+XL_S
LEPSKITK+XL_L
AMKQVAGTMK+XL_S
AMKQVAGTMK+XL_L
B_b_ion
B_y_ion
m/z
intensity
0
200
400
600
800
1000
100
300
y8++
y7+
y8+
prec
